# Recent advances in the management and understanding of macular degeneration

**DOI:** 10.12688/f1000research.10998.1

**Published:** 2017-04-20

**Authors:** Sepehr Bahadorani, Michael Singer

**Affiliations:** 1 University of Texas Health Science Center at San Antonio, San Antonio, TX, USA; 2 Medical Center Ophthalmology Associates, San Antonio, TX, USA

**Keywords:** macular degeneration, stem cell, lampalizumab

## Abstract

Current management of age-related macular degeneration (AMD) is directed at intravitreal injection of vascular endothelial growth factor (VEGF) inhibitors for the treatment of wet AMD and supplementation with oral antioxidants for the treatment of dry AMD. In this article, we will review recent clinical trials for the treatment of dry and wet AMD.

## Introduction

Age-related macular degeneration (AMD) is a common cause of irreversible blindness in elderly populations of the Western countries. The disorder is characterized by the appearance of drusen in the macula followed by geographic atrophy or choroidal neovascularization (CNV) ^1^. The exact etiology of AMD remains unknown, yet it is thought that environmental factors as well as mutations in genes of various biochemical pathways including lipid transport and metabolism, the complement cascade, remodeling of the retinal extracellular collagen matrix, and the angiogenesis pathway may contribute to the development of AMD ^2^.

Two advanced forms of AMD have been identified: 1) “dry” or atrophic AMD, which accounts for 85–90% of AMD cases and presents with atrophy of the retinal pigment epithelium (RPE) with subsequent progressive visual loss, and 2) “wet” or neovascular AMD, accounting for 10–15% of cases and characterized by the growth of new blood vessels from the choroid into the Bruch’s membrane with subsequent leakage and bleeding that disrupt the normal architecture of the photoreceptor-RPE complex and, ultimately, lead to scar formation ^3^.

While different antioxidants and oral agents are used in various trials to delay the progression of atrophic AMD, intravitreal vascular endothelial growth factor (VEGF) inhibitors remain the mainstay of treatment for neovascular AMD ^4, 5^. The goal of this review is to focus on recent treatments that are being developed for the treatment of AMD.

## Clinical trials

The list of clinical trials for this review was obtained from PubMed searches using the following keywords: “macular degeneration”, “randomized”, and “trial”. Additionally, the content of currently published review articles on different clinical trials of AMD were studied for the inclusion of relevant trials ^6– 8^. Finally, applicable trials presented at different meetings including the Association for Research in Vision and Ophthalmology (ARVO) and the American Academy of Ophthalmology’s subspecialty day were also included.

## Treatments for dry age-related macular degeneration

### Lampalizumab

The complement pathway is an important component of the host-defense system, yet it must be tightly regulated to prevent tissue inflammation and damage. Lampalizumab (FCFD4514S) is a humanized IgG Fab fragment that inhibits complement factor D, the rate-limiting enzyme in the alternative pathway of the complement cascade, which has been implicated in the pathogenesis of geographic atrophy ^9, 10^. Indeed, polymorphisms in gene regulators of the alternative complement pathway are associated with increased risk of AMD ^11^.

An earlier phase Ia trial showed that the administration of single-dose intravitreal lampalizumab was safe and well tolerated in patients with geographic atrophy ^12^. Following this safety trial, the MAHALO phase Ib/II trial (NCT01229215) demonstrated that monthly treatment with lampalizumab in patients with geographic atrophy reduces the rate of lesion progression by about 20% at month 18 ^13^. Presently, there are two phase III trials underway to evaluate the long-term effects of lampalizumab intravitreal injections in patients with geographic atrophy (NCT02745119 and NCT02247531).

### Stem cell transplantation

Stem cells have the potential to replace damaged cells and thus carry wide applications in regenerative therapeutics and the field of neurodegenerative disorders ^14^. Recent studies show that subretinal transplantation of human embryonic stem cell (hESC)-derived RPE could improve, or at least stabilize, visual acuity in patients with dry AMD (n=2) and Stargardt macular dystrophy (n=2). One patient with dry AMD and initial best-corrected visual acuity (BCVA) of one letter maintained two letters read by the end of their 1-year visit. The second dry AMD patient with initial BCVA of 20/320 (25 ETDRS letters) improved to 20/200 (34 ETDRS letters) at their 1-year visit and the central scotoma tested by Goldmann perimetry diminished in intensity. Enhanced subretinal pigmentation with localized black clumps, development of epiretinal membrane without macular puckering, corneal abrasion, elevated intraocular pressure, and subretinal hemorrhage were reported events following the transplantation procedure. However, there was no evidence of adverse proliferation, cancer development, or ectopic tissue formation after 1 year of therapy ^15^. These findings provide increasing hopes of vision recovery using stem cell transplantation in patients with advanced dry AMD. Presently, there is an additional multicenter clinical trial underway with encouraging results at 1 month that will address the safety of umbilical cord-derived stem cells in patients with geographic atrophy (NCT01226628).

### AAV2.sCD59 gene therapy

CD59 complement factor is a naturally occurring membrane-bound inhibitor of the membrane attack complex (MAC), an immune protein that mediates cell lysis by the formation of plasma membrane pores ^16, 17^. Indeed, there is increased abundance of MAC in the choriocapillaris of aging retina and AMD patients ^18, 19^, prompting investigators to evaluate the role of CD59 in the progression of AMD. Animal studies show that intraocular delivery of an adeno-associated virus vector (AAV2) that expresses the soluble form of membrane-independent CD59 (sCD59) reduces CNV, vascular leakage, and retinal ganglion cell death in a mouse model of diabetic retinopathy ^20^. Hemera Biosciences has recently obtained FDA approval to carry out a phase I gene therapy trial to evaluate the safety of MAC inhibition via AAV2.sCD59 vectors in participants with dry AMD.

### Photobiomodulation

Photobiomodulation is the procedure through which visible to near-infrared light is applied to cells to produce beneficial effects. The results of a recent study on 42 patient with dry AMD show that application of multi-wavelength light composed of yellow (590 nm), red (670 nm) and near-infrared (790 nm) for a period of 3 weeks results in significant improvement in BCVA, contrast sensitivity, and the drusen size ^21^. Presently, participants are being recruited for further evaluation of photobiomodulation as a treatment of dry AMD [NCT02725762].

### Brimonidine

Brimonidine is a selective alpha-2 receptor adrenergic agonist that, in addition to its intraocular pressure (IOP)-lowering effects, carries neuroprotective properties that are thought to be linked to reduced accumulation of extracellular glutamate and blockade of NMDA receptors ^22^. Currently, Allergan has sponsored a phase II clinical trial to evaluate the safety and efficacy of a brimonidine tartrate intravitreal implant in the treatment of patients with dry AMD. The initial results of the study involving 113 participants demonstrate that, in comparison to the sham arm, administration of low (200 µg) and high (400 µg) dose inserts of 22 gauge sustained-release brimonidine at baseline and at month 6 results in a statistically significant reduction in the size of geographic atrophy for both low (by 18%) and high (by 27%) dose implants. No significant adverse effects have been reported for either inserts (NCT00658619). A second phase II trial is underway to investigate the efficacy and safety of a new 25 gauge brimonidine insert (400 µg) administered to study eyes on day 1 and every 3 months until month 21 (NCT02087085).

### Tetracyclines

Minocycline is a tetracycline derivative with neuroprotective properties that are thought to be attributed to its caspase inhibitory functions ^23^. Furthermore, *in vitro* studies demonstrate that minocycline protects primary human RPE cells against oxidative stress ^24^. Presently, there are ongoing phase II trials that are evaluating the effects of oral minocycline (100 mg, twice daily) and doxycycline (40 mg, daily) in the treatment of AMD patients with geographic atrophy (NCT02564978 and NCT01782989).

## Treatments for wet age-related macular degeneration

### Anti-vascular endothelial growth factor agents

Abicipar pegol (MP0112), a designed ankyrin repeat protein (DARPin), is a genetically engineered antibody mimetic protein that is used via intravitreal injection to target VEGF (NCT01086761). Results of initial studies demonstrate that MP0112 decreases mean retinal thickness and leakage area despite ocular inflammation in patients with neovascular AMD ^25^. Currently, two independent phase III studies are enrolling (NCT02462486 and NCT02462928).

Squalamine is another anti-VEGF agent that has been used for the treatment of wet AMD. Ohr Pharmaceutical Inc. has presented the results from the IMPACT phase II study, which evaluated the effects of combination therapy of 0.2% squalamine lactate ophthalmic solution administered twice daily with monthly ranibizumab intravitreal injections in patients with wet AMD (NCT02511613). Squalamine lactate is known to inhibit angiogenesis by entering into activated endothelial cells through caveolae followed by binding to and manipulating calmodulin to block angiogenesis ^26^. Data from the IMPACT study demonstrate that, after 9 months of treatment, patients with combination therapy of ranibizumab and squalamine lactate had a mean gain of 11 letters versus a gain of 5 letters in the ranibizumab monotherapy arm. Furthermore, at 9 months, 44% of the patients receiving combination therapy achieved a ≥3 line vision gain as compared to 29% in the ranibizumab monotherapy arm ^27^. Presently, a phase III trial of squalamine lactate is underway (NCT02727881).

Single-chain antibody fragment VEGF inhibitor RTH258 has been tested in a phase I/II trial in treatment-naive patients with subfoveal CNV secondary to AMD. The trial demonstrates non-inferiority of RTH258 in comparison to ranibizumab in mean change of central subfield thickness from baseline to month 1. Likewise, changes in BCVA were comparable between RTH258 and ranibizumab ^28^. Phase III studies are now underway to compare the efficacy and safety of RTH258 to aflibercept (NCT02307682 and NCT02434328).

Finally, DS7080a (Daiichi Sankyo Inc.) is another angiogenesis-inhibiting monoclonal antibody that is being evaluated in a phase I clinical trial for the treatment of wet AMD (NCT02530918).

### Small molecules

PAN-90806 is an alternative topical anti-VEGF eye drop with promising outcomes that is being used by PanOptica Inc. for the treatment of wet AMD (NCT02022540). Currently, the medication is being reformulated and will go back into clinical trials ^29^.

### Anti-platelet-derived growth factor agents

Platelet-derived growth factor (PDGF) promotes pericyte recruitment, endothelial proliferation, and angiogenesis ^30, 31^. In light of the role of PDGF in neovascularization, Ophthotech has developed E10030 (Fovista®), a PDGF antagonist, for the treatment of patients with wet AMD. In a phase IIb clinical trial, participants were randomized to ranibizumab treatment in combination with E10030 0.3 mg, E10030 1.5 mg, or sham. Nevertheless, despite favorable visual acuity outcomes for the E10030 1.5 mg arm ^32^, the phase III trial of E10030 has been terminated (NCT01944839). In December 2016, Ophthotech announced that the primary endpoint of mean change in visual acuity at 12 months was not achieved in phase III clinical trials that were investigating the superiority of Fovista and Lucentis combination therapy compared to Lucentis monotherapy for the treatment of wet AMD ^33^. Likewise, the phase II CAPELLA trial of intravitreal REGN2176-3 (Regeneron Pharmaceuticals Inc.), an anti-PDGF receptor beta antibody, failed to reach its primary endpoint in neovascular AMD ^34^.

### Anti-angiopoietin agents

In addition to VEGF molecules, angiopoietins play an essential role in the formation of new blood vessels ^35^. A recent phase I trial has investigated the effects of RG7716, an anti-VEGF/anti-Ang2 antibody, in patients with wet AMD who had persistent CNV despite three or more intravitreal anti-VEGF treatments in the preceding 6 months, with the last treatment applied at least 4 weeks prior to enrolment. The results of this trial confirm the safety profile of RG7716 with promising improvements in BCVA as well as central subfield thickness ^36^. Presently, a phase II trial is underway to investigate the effects of RG7716 in larger patient populations (NCT02484690).

### rAAV.sFLT-1 gene therapy

In comparison to intravitreal injection of anti-VEGF agents, the application of recombinant adeno-associated virus (rAAV) vector gene therapy allows for longer duration of reversing the AMD pathologic processes. Soluble fms-like tyrosine kinase-1 (sFLT-1) is a naturally occurring anti-angiogenic protein that confers protection against CNV, where subretinal injection of rAAV.sFLT-1 vector in a mouse model is shown to reduce fluorescein leakage from the retinal vessels and lower the number of aberrant vessels invading the outer nuclear layer ^37^. The gene therapy vector is also safe and well tolerated in humans ^38^.

Recently, a phase IIa randomized controlled trial investigated the effects of subretinal administration of rAAV.sFLT-1 gene therapy vector in patients with active wet AMD. All patients (n=32) received ranibizumab injections at baseline and week 4, after which, following a core vitrectomy at day 7, patients in the gene therapy group (n=21) received subretinal injection of 100 μL rAAV.sFLT-1 vector (1 × 10 ^11^ vg). In the rAAV.sFLT-1 group, BCVA improved by a median of one ETDRS letter from baseline compared to a median of five letters’ loss in the control group at week 52. Furthermore, 11 (52.4%) rAAV.sFLT-1-treated patients received two or fewer ranibizumab retreatments and three (14.3%) participants achieved notable BCVA gains of ≥15 letters versus the control arm, in which 10 of 11 patients (90.9%) received more than two ranibizumab retreatments and none achieved a gain of ≥15 letters. While a larger sample size is required to obtain meaningful statistical outcomes, the results of this study support the role of ocular gene therapy as a potential approach for the long-term treatment of wet AMD ^39^. Currently, rAAV is being formulated for the delivery of alternative genes.

## Future directions

The future of dry AMD treatments are trending towards long-term vision preservation. For wet AMD, it is to decrease treatment burden as well to achieve better visual results. This is being done by targeting other sites in the pathway leading to the development of wet AMD.

## References

[1] Gheorghe A Mahdi L Musat O: AGE-RELATED MACULAR DEGENERATION. *Rom J Ophthalmol.* 2015;59(2):74–7. 26978865 PMC5712933

[2] Francis PJ Klein ML: Update on the role of genetics in the onset of age-related macular degeneration. *Clin Ophthalmol.* 2011;5:1127–33. 10.2147/OPTH.S11627 21887094 PMC3162292

[3] Bhutto I Lutty G: Understanding age-related macular degeneration (AMD): relationships between the photoreceptor/retinal pigment epithelium/Bruch’s membrane/choriocapillaris complex. *Mol Aspects Med.* 2012;33(4):295–317. 10.1016/j.mam.2012.04.005 22542780 PMC3392421

[4] Schmidl D Garhofer G Schmetterer L: Nutritional supplements in age-related macular degeneration. *Acta Ophthalmol.* 2015;93(2):105–21. 10.1111/aos.12650 25586104

[5] Villegas VM Aranguren LA Kovach JL: Current advances in the treatment of neovascular age-related macular degeneration. *Expert Opin Drug Deliv.* 2017;14(2):273–82. 10.1080/17425247.2016.1213240 27434329

[6] Leung E Landa G: Update on current and future novel therapies for dry age-related macular degeneration. *Expert Rev Clin Pharmacol.* 2013;6(5):565–79. 10.1586/17512433.2013.829645 23971874

[7] Singer M: Advances in the management of macular degeneration. *F1000Prime Rep.* 2014;6:29. 10.12703/P6-29 24860651 PMC4017905

[8] Taskintuna I Elsayed ME Schatz P: Update on Clinical Trials in Dry Age-related Macular Degeneration. *Middle East Afr J Ophthalmol.* 2016;23(1):13–26. 10.4103/0974-9233.173134 26957835 PMC4759891

[9] Katschke KJ Jr Wu P Ganesan R: Inhibiting alternative pathway complement activation by targeting the factor D exosite. *J Biol Chem.* 2012;287(16):12886–92. 10.1074/jbc.M112.345082 22362762 PMC3339934

[10] Le KN Gibiansky L van Lookeren Campagne M: Population Pharmacokinetics and Pharmacodynamics of Lampalizumab Administered Intravitreally to Patients With Geographic Atrophy. *CPT Pharmacometrics Syst Pharmacol.* 2015;4(10):595–604. 10.1002/psp4.12031 26535160 PMC4625864

[11] van Lookeren Campagne M Strauss EC Yaspan BL: Age-related macular degeneration: Complement in action. *Immunobiology.* 2016;221(6):733–9. 10.1016/j.imbio.2015.11.007 26742632

[12] Do DV Pieramici DJ van Lookeren Campagne M: A phase ia dose-escalation study of the anti-factor D monoclonal antibody fragment FCFD4514S in patients with geographic atrophy. *Retina.* 2014;34(2):313–20. 10.1097/IAE.0b013e3182979ddd 23842100

[13] Roche. Reference Source

[14] Singh S Srivastava A Srivastava P: Advances in Stem Cell Research- A Ray of Hope in Better Diagnosis and Prognosis in Neurodegenerative Diseases. *Front Mol Biosci.* 2016;3:72. 10.3389/fmolb.2016.00072 27878120 PMC5099954

[15] Song WK Park KM Kim HJ: Treatment of macular degeneration using embryonic stem cell-derived retinal pigment epithelium: preliminary results in Asian patients. *Stem Cell Reports.* 2015;4(5):860–72. 10.1016/j.stemcr.2015.04.005 25937371 PMC4437471

[16] Cashman SM Ramo K Kumar-Singh R: A non membrane-targeted human soluble CD59 attenuates choroidal neovascularization in a model of age related macular degeneration. *PLoS One.* 2011;6(4):e19078. 10.1371/journal.pone.0019078 21552568 PMC3084256

[17] Ricklin D Hajishengallis G Yang K: Complement: a key system for immune surveillance and homeostasis. *Nat Immunol.* 2010;11(9):785–97. 10.1038/ni.1923 20720586 PMC2924908

[18] Chirco KR Tucker BA Stone EM: Selective accumulation of the complement membrane attack complex in aging choriocapillaris. *Exp Eye Res.* 2016;146:393–7. 10.1016/j.exer.2015.09.003 26368849 PMC4788569

[19] Mullins RF Schoo DP Sohn EH: The membrane attack complex in aging human choriocapillaris: relationship to macular degeneration and choroidal thinning. *Am J Pathol.* 2014;184(11):3142–53. 10.1016/j.ajpath.2014.07.017 25204844 PMC4215023

[20] Adhi M Cashman SM Kumar-Singh R: Adeno-associated virus mediated delivery of a non-membrane targeted human soluble CD59 attenuates some aspects of diabetic retinopathy in mice. *PLoS One.* 2013;8(10):e79661. 10.1371/journal.pone.0079661 24167638 PMC3805538

[21] Merry GF Munk MR Dotson RS: Photobiomodulation reduces drusen volume and improves visual acuity and contrast sensitivity in dry age-related macular degeneration. *Acta Ophthalmol.* 2016. 10.1111/aos.13354 27989012 PMC5484346

[22] Doozandeh A Yazdani S: Neuroprotection in Glaucoma. *J Ophthalmic Vis Res.* 2016;11(2):209–20. 10.4103/2008-322X.183923 27413504 PMC4926571

[23] Thomas M Le WD: Minocycline: neuroprotective mechanisms in Parkinson’s disease. *Curr Pharm Des.* 2004;10(6):679–86. 10.2174/1381612043453162 14965330

[24] Kernt M Thiele S Hirneiss C: Altersbedingte Makuladegeneration: die Rolle von Licht bei der Entstehung degenerativer Veranderungen im menschlichen RPE und moglicher Zell-Schutz durch Minocyclin. *Klin Monbl Augenheilkd.* 2011;228(10):892–9. 10.1055/s-0029-1245892 21432767

[25] Souied EH Devin F Mauget-Faÿsse M: Treatment of exudative age-related macular degeneration with a designed ankyrin repeat protein that binds vascular endothelial growth factor: a phase I/II study. *Am J Ophthalmol.* 2014;158(4):724–732.e2. 10.1016/j.ajo.2014.05.037 24907435

[26] Connolly B Desai A Garcia CA: Squalamine lactate for exudative age-related macular degeneration. *Ophthalmol Clin North Am.* 2006;19(3):381–91, vi. 16935213 10.1016/j.ohc.2006.05.003

[27] IMPACT Study. Reference Source

[28] Holz FG Dugel PU Weissgerber G: Single-Chain Antibody Fragment VEGF Inhibitor RTH258 for Neovascular Age-Related Macular Degeneration: A Randomized Controlled Study. *Ophthalmology.* 2016;123(5):1080–9. 10.1016/j.ophtha.2015.12.030 26906165

[29] Cousins SW: PAN-90806-a Novel Topical Treatment for Neovascular AMD. Reference Source

[30] Gianni-Barrera R Bartolomeo M Vollmar B: Split for the cure: VEGF, PDGF-BB and intussusception in therapeutic angiogenesis. *Biochem Soc Trans.* 2014;42(6):1637–42. 10.1042/BST20140234 25399582

[31] Battegay EJ Rupp J Iruela-Arispe L: PDGF-BB modulates endothelial proliferation and angiogenesis *in vitro* via PDGF beta-receptors. *J Cell Biol.* 1994;125(4):917–28. 10.1083/jcb.125.4.917 7514607 PMC2120083

[32] Jaffe GJ Ciulla TA Ciardella AP: Dual Antagonism of PDGF and VEGF in Neovascular Age-Related Macular Degeneration: A Phase IIb, Multicenter, Randomized Controlled Trial. *Ophthalmology.* 2017;124(2):224–34. 10.1016/j.ophtha.2016.10.010 28029445

[33] http://www.businesswire.com/news/home/20161211005098/en/Ophthotech-Announces-Results-Pivotal-Phase-3-Trials.

[34] CAPELLA study. Reference Source

[35] Yancopoulos GD Davis S Gale NW: Vascular-specific growth factors and blood vessel formation. *Nature.* 2000;407(6801):242–8. 10.1038/35025215 11001067

[36] Chakravarthy U Schwab D Cech P: The novel bispecific monoclonal anti-VEGF/anti-Ang2 antibody RG7716 shows promise in wet age-related macular degeneration patients with suboptimal response to prior anti-VEGF monotherapy.ARVO 2016 Annual Meeting Abstracts.

[37] Lai CM Estcourt MJ Wikstrom M: rAAV.sFlt-1 gene therapy achieves lasting reversal of retinal neovascularization in the absence of a strong immune response to the viral vector. *Invest Ophthalmol Vis Sci.* 2009;50(9):4279–87. 10.1167/iovs.08-3253 19357358

[38] Rakoczy EP Lai CM Magno AL: Gene therapy with recombinant adeno-associated vectors for neovascular age-related macular degeneration: 1 year follow-up of a phase 1 randomised clinical trial. *Lancet.* 2015;386(10011):2395–403. 10.1016/S0140-6736(15)00345-1 26431823

[39] Constable IJ Pierce CM Lai CM: Phase 2a Randomized Clinical Trial: Safety and Post Hoc Analysis of Subretinal rAAV.sFLT-1 for Wet Age-related Macular Degeneration. *EBioMedicine.* 2016;14:168–75. 10.1016/j.ebiom.2016.11.016 27865764 PMC5161436

